# The effects of preoperative alcohol, tobacco, and psychological stress on postoperative complications: a prospective observational study

**DOI:** 10.1186/s12871-021-01456-w

**Published:** 2021-10-13

**Authors:** Yoshinori Myoga, Haruhiko Manabe, Yoneatsu Osaki

**Affiliations:** 1 Department of Anesthesiology, Kitakyushu Municipal Medical Center, 2-1-1 Bashaku, Kokurakita-ku, Kitakyushu, Fukuoka, 802-0077 Japan; 2 Division of Environmental and Preventive Medicine, Department of Social Medicine, Faculty of Medicine, Tottori University, 86 Nishi-cho, Yonago, Tottori, 683-8503 Japan

**Keywords:** Alcohol, Mental stress, Postoperative complications, Psychological stress, Tobacco

## Abstract

**Background:**

Postoperative complications occur frequently, despite progress in anesthetic pharmacology and surgical techniques. Although habits, such as alcohol and tobacco use, and mental health have been studied individually as modifying factors, few studies have examined the relationship between multiple lifestyle choices and postoperative complications in patients undergoing surgery. Hence, this study aimed to investigate the associations between unhealthy lifestyle choices and postoperative complications.

**Methods:**

We included 730 patients who underwent surgery in our department between March 2015 and April 2016. Participants completed preoperative questionnaires, including the Alcohol Use Disorders Identification Test, Fagerström Test for Nicotine Dependence, and tests for psychological stress (6-item Kessler Psychological Distress Scale; Hospital Anxiety and Depression Scale). Multivariable logistic analysis was used to analyze the association of preoperative drug dependence and psychological stress with postoperative complications.

**Results:**

Of the 721 cases analyzed, 461 (64%) were women. The median age of patients was 62 years (interquartile range: 48–71). At the time of surgical decision-making, 429 out of 710 respondents (60%) had a drinking habit, and 144 out of 693 respondents (21%) had a smoking habit during the preceding year. Seventy-nine patients had developed complications. Multivariable analysis revealed that old age (*p* = 0.020), psychological stress (*p* = 0.041), and longer anesthesia time (*p* < 0.001) were significantly associated with postoperative complications. Drinking or smoking variables were not associated with postoperative complications.

**Conclusions:**

Preoperative psychological stress, as evaluated with the 6-item Kessler Psychological Distress Scale, is associated with the risk of postoperative complications.

## Background

Despite advances in surgical procedures and anesthetic pharmacology, postoperative mortality rates of 0.5–4.0%, mostly attributed to postoperative complications, have been reported ininternational multicenter cohort studies [1, 2]. Postoperative complications affect 16.8% of elective surgeries, and between 30 and 60% in major abdominal surgeries [1, 3]; the patient’s associated risk factors are typically old age, multiple comorbidities, frail, and unhealthy lifestyle and behavioral factors [4]. Although lifestyle factors, such as alcohol and tobacco use, reduce psychophysiological resilience and delay postoperative early recovery, those factors, including psychological stress, can be mitigated preoperatively [5–8]. By doing so, we can enhance the physiological and psychological resilience of patients and reduce postoperative complications [9].

Unhealthy lifestyle factors frequently cluster in the same individual and hence multimodal interventions for modifiable risk factors are warranted to enhance resilience in the time-pressured preoperative window [10, 11]. However, lifestyle risks on surgical outcomes are often studied separately, to our knowledge, the association of multiple lifestyle risk factors with postoperative complications has not been studied in-depth in the surgical patient population [5–8]. Predicting the susceptibility of individuals to postoperative complications and assessing the impact of smoking, alcohol, psychological stress, and their interactions on postoperative complications is important to prevent postoperative mortality.

This study assessed the effects of preoperative alcohol and tobacco use, and psychological stress on postoperative complications.

## Methods

### Study sample

Following the Declaration of Helsinki, this study was conducted after obtaining approval from the Ethics Committee of the Kitakyushu Municipal Medical Center and Faculty of Medicine, Tottori University (Approval No.: 2662). The study participants were adult patients who underwent planned non-cardiac surgery with intubation under general anesthesia at the Kitakyushu Municipal Medical Center between March 2015 and April 2016.

A total of 730 participants completed the questionnaires to evaluate preoperative psychological stress, drinking/smoking habits, and alcohol/nicotine dependence. Inclusion criteria included patients between categories I and III in the American Society of Anesthesiologists physical status classification (ASA-PS), who were at least 20 years of age. Exclusion criteria included the inability to complete to the questionnaire due to dementia or dysgraphia, as judged by the anesthesiologists.

### Procedure

Questionnaires were given to patients at the preoperative consultation (1-3 days before surgery). Anesthesiologists (YM and HM) provided a verbal explanation of the study using the patient’s manual, including the study details, such as purpose, methods, the right to refuse participation, and contact information for questions and complaints related to the study (Opt-in and Opt-out). Patients provided completed and signed questionnaires to attending anesthesiologists, implying consent for the study.

We analyzed how drinking/smoking habits, the degree of dependence, and the degree of psychological stress were related to postoperative complications within 30 days of surgery. The patients’ background, surgical, and anesthesia data were obtained from the electronic medical records and preoperative questionnaires. We also investigated the duration of hospitalization and in-hospital death as secondary outcomes. Postoperative complications included those that met the Japan Clinical Oncology Group (JCOG) postoperative complications criteria, as well as those that required additional treatment, such as hyperactive delirium, asthma, and catheter infections.

### Measurements (covariates)

We assessed factors known to affect postoperative complications to include as covariates, including age, sex, body mass index (BMI), ASA-PS (as determined by attending anesthesiologists), Preoperative use of psychotropics (hypnotics, anxiolytics, and antidepressants), anesthesia time/surgical time, and the Surgical Apgar score (sAs, collected from the electronic anesthesia records by the first author). Details of each evaluation tool are outlined below.

#### Alcohol screening in the year prior to surgery

The Alcohol Use Disorder Identification Test (AUDIT) is a self-administered screening test that was developed by the WHO in the 1980s to evaluate alcohol consumption, dependence, and problematic drinking [12]. The AUDIT includes 10 items and can be used across various ethnicities and cultures. Respondents are asked to answer 10 questions regarding alcohol use in the preceding year; items 1–3 assess the amount and frequency of alcohol consumption, items 4–6 assess the degree of dependence, and items 7–10 assess problematic drinking, with a total score of 0–40 points. The cutoff value for problematic drinking is 8 points or higher.

#### Tobacco screening in the year prior to surgery

The Fagerström Test for Nicotine Dependence (FTND) is a modified version of the Fagerström Tolerance Questionnaire and was developed by Heatherton et al. to assess the need for nicotine replacement therapy in the treatment of withdrawal symptoms among smokers who are trying to quit [13]. This simplified test contains six items that assess tobacco consumption and the degree of dependence. Each item has a varying degree of importance (points), and scores are summed to yield a total score of 10. The degree of dependence is categorized as follows: Low dependence (0–2 points), medium dependence (3–6 points), and high dependence (7–10 points) [14]. The cutoff for nicotine dependence was 3 points or higher.

#### Mental health screening 30 days prior to surgery

In the medical field, the Hospital Anxiety and Depression Scale (HADS) is a well-established self-administered 14-item scale that has two subscales that assess anxiety (7 items) and depression (7 items). The HADS was developed by Zigmond and Snaith in 1983 to evaluate the degree of anxiety and depression in non-psychiatric hospitals and clinics [15]. Items related to physical symptoms, such as dizziness, headache, and low appetite were not included as they are not depression-specific symptoms. Respondents rated each HADS item on a 4-point scale (0–3 points), whereby a score of 0 is the most positive, and a score of 3 is the most negative. A higher HADS score represents a greater risk of anxiety and depressive disorders. A score of 11–19 points indicates adjustment disorder, and a score of 20 or more indicates major depressive disorder [16].

Meanwhile, the 6-item Kessler Psychological Distress Scale (K6) is a simplified version of the 10-item version of the scale and evaluates nonspecific psychological stress in workplaces and the field of epidemiology [17], hence K6 can assess the overall psychological stress, such as mood and anxiety disorders, but not depression. Arnaud et al. reported that the K6 is a convenient tool with a reliability that is equivalent to that of the K10 [18]. In this study, we used the Japanese version of the K6, which includes items 2, 4, 5, 8, 9, and 10 of the K10. Each K6 item is scored on a scale of 1 to 5 (1: never; 2: rarely; 3: some of the time; 4: most of the time; 5: all the time), with a total score 0f 0–24 points. We defined that a score of 8 points or higher was considered as indicative of psychological stress [19].

#### The preoperative severity of patient comorbidity (ASA-PS)

The ASA-PS was developed by Meyer Saklad in 1941 [20]. The goal of the ASA-PS classification is to assess the overall health status and comorbidities of preoperative patients. Items are graded on a 6-point scale crudely, whereby a higher score indicates a higher risk of perioperative complications, 30-day readmissions, and longer hospitalization [20, 21]. This is a subjective classification that is completed by the evaluator (an attending anesthesiologist) and does not consider surgical invasiveness, the method of anesthesia, or other intraoperative factors. Given that this is only a preoperative evaluation of a patient, we also used the sAs as an objective measure of intraoperative invasiveness in this study.

#### The intraoperative surgical and anesthetic invasiveness (sAs)

Following the Apgar score in obstetrics, Gawande et al. proposed the sAs in 2007, which has a maximum score of 10 points [22]. This total score is calculated by quantifying and scoring three parameters from intraoperative anesthesia records, including lowest mean blood pressure, lowest heart rate, and amount of blood loss. The amount of blood loss is an indicator of surgical complexity and the surgeon’s performance, and changes in heart rate and blood pressure reflect the patient’s physiological state and the appropriateness of anesthetic management. This scoring system effectively predicts the postoperative 30-day mortality rate and postoperative complications, for both general surgery and vascular surgery [23]. A maximum sAs (10 points) indicates the most appropriate intraoperative management.

### Assessment of postoperative complications

The main outcome of this study was the occurrence of 30-day postoperative complications that required additional treatment or extended hospitalization. All complications were defined by the adverse event (AE) terms from the JCOG postoperative complications criteria. Those that were not included in the AE terms were classified as “others” (e.g., delirium, bronchial asthma, and catheter infections, among others).

#### JCOG postoperative complications criteria version 2

This measure is based on the original article describing the Clavien–Dindo classification of surgical complications (2004) [24], which has been frequently used to objectively assess procedural skills and 72 postoperative complications (73 items including “Others”) [25]. The AE terms and each of their grading details were standardized from the Clavien–Dindo classification, with the approval of the JCOG operational committee. Version 2 (2013) is the most recent. Furthermore, AE terms such as pneumonia, ileus, anastomotic leak, wound infection, and postoperative hemorrhage were graded, from grade I to grade V (death). This study did not take the severity of complications into consideration.

### Statistical analysis

For statistical testing of medians, the Mann–Whitney U test was used for the analysis for the comparison of patients with and without postoperative complications. For statistical testing of proportions, the chi-squared test was used to test the difference in proportion for the comparison of patients with and without postoperative complications. When the expected numbers for the chi-squared test were small, we used a Fisher’s exact test. We conducted multiple logistic regression analysis. The dependent variable was postoperative complications, and independent variables were age, sex, BMI, ASA-PS, Preoperative use of psychotropics, sAs, anesthesia time, drinking and smoking habits (AUDIT, FTND), HADS, and K6. Model 1 included improved drinking/smoking habits after surgical decision-making as a covariate, and Model 2 included the degree of alcohol/nicotine dependence at the time of surgical decision-making as a covariate. A male and female subset of Model 2 (Model 3 and 4, respectively) was also created. Taking multicollinearity into consideration, for a correlation of 0.4 and above, one of the two variables was removed. We used the c-statistic (area under the curve [AUC]) and accuracy as indicators of the model’s predictive precision. JMP version 12 (from SAS) was used for modeling, with a significance cutoff of *p* < 0.05.

We assigned zero points to missing items in the AUDIT, FTND, K6, and HADS if more than 75% of all questions had been answered. The questionnaires with the following missing responses were excluded from the analysis: AUDIT responses with items 1–3 missing (questions related to the amount and frequency of drinking), FTND responses with item 4 missing (number of cigarettes smoked per day), and responses with 25% or more missing items (3 or more items in the AUDIT, 2 or more items in the FTND and K6, and 4 or more items in the HADS).

## Results

A final total of 721 patients provided the questionnaires and were included in the analysis. Table 1 shows the type of surgery and the incidence of postoperative complications within 30 days of surgery. Of the 461 (64%) women, 208 (29%) were breast surgery cases. The rate of postoperative complication development was 11% in 79 cases, which included anastomotic leak/peritonitis (*n* = 11), wound infection/dehiscence (*n* = 8), postoperative hemorrhage (*n* = 5), and myocardial infarction (n = 1). The duration of postoperative hospitalization was longer in patients with postoperative complications than those without complications (*p* < 0.001).

**Table 1:** Details of the surgery and postoperative complications

**Type of surgery**	**n (%)**
Breast surgery	209 (29.0)
Gastrointestinal surgery	129 (17.9)
Orthopedic surgery	96 (13.3)
Otolaryngology surgery	66 (9.2)
Abdominal surgery for gynecology	51 (7.1)
Cholecystectomy, staging laparoscopy, stoma construction/closure	33 (4.5)
Thoracic surgery	29 (4.0)
Thyroid/parathyroid surgery	27 (3.7)
Hepatic surgery	23 (3.2)
Abdominal surgery for urology	19 (2.6)
Pancreatic surgery	17 (2.4)
Transvaginal/urethral surgery	8 (1.1)
Craniotomy	3 (0.4)
Hernia repair	2 (0.3)
Others (minor surgery)	9 (1.3)
**Postoperative complications**	**n (%)**
Anastomotic leak/Peritonitis	11 (1.5)
Pancreatic fistula/Gallbladder fistula	9 (1.2)
Wound infection/dehiscence	8 (1.1)
Intra-abdominal abscess	8 (1.1)
Ileus/Gastroesophageal reflux disease	6 (0.8)
Postoperative hemorrhage	5 (0.7)
Atrial Fibrillation	4 (0.6)
Pneumonia/Atelectasis	3 (0.4)
Anastomotic stenosis	3 (0.4)
Pulmonary fistula/Pleural effusion	2 (0.3)
Enterovesical fistula/Rectalvaginal fistula	2 (0.3)
Ischemic heart disease	1 (0.1)
Implant infection	1 (0.1)
Urinary retention	1 (0.1)
Other	
Hyperactive delirium	8 (1.1)
Bronchial asthma	2 (0.3)
Infection of Infusion line	2 (0.3)
Herpes zoster, influenza, pustulosis, diverticulitis	4 (0.6)
In-hospital death (137 days after surgery)	1
In-hospital death after readmission	1
Reoperation within 30 days of surgery	12
Readmission	4
Length of stay after surgery (days)	9 (6–13)

Details of postoperative complications in 721 patients within 30 days of surgery. A total of 80 complications were found in 79 out of 721 patients (11%). One patient experienced both atrial fibrillation and pleural effusion

At the time of the surgical decision, 429 out of 710 respondents (60%) had a drinking habit, and 144 out of 693 respondents (21%) had a smoking habit in the preceding year. Within those with either drinking or smoking habits in the preceding year, 62 and 71% of patients, respectively, had either reduced or quit their habits by the day before surgery (Table 2). We applied univariable analysis of each factor against sex and postoperative complications. The duration of surgery and anesthesia were significantly longer in patients with postoperative complications (*p* < 0.001).

**Table 2:** Association of postoperative complications and patient characteristics by sex

	All (721)	Male (260)			Female (461)		
		Postoperative complications			Postoperative complications		
		No (215)	Yes (45)	*p* value	No (427)	Yes (34)	p value
Age (years)	62 (48–71)	65 (52–72)	66 (59–72)	0.27	57 (47–69)	67(62–73)	0.001*
BMI (kg/m^2^)	23 (21–25)	24 (22–27)	23 (22–25)	0.18	22 (20–25)	22 (19–24)	0.39
ASA-PS I/II/III	212/483/26	30/175/10	2/41/2	0.21	172/242/13	8/25/1	0.15
Preoperative use of psychotropics	85/693 (12%)	23/206 (11%)	5/45 (11%)	1.00	51/409 (12%)	6/33 (18%)	0.41
Surgical time (minutes)	125 (81–201)	104 (66–227)	262 (186–344)	p < 0.001*	118 (85–161)	196 (131–294)	*p* < 0.001*
Anesthesia time (minutes)	176 (134–254)	173 (107–282)	305 (236–443)	p < 0.001*	168 (135–218)	239 (179–359)	p < 0.001*
sAs	8 (7–9)	8 (7–8)	7 (6–8)	0.039*	8 (7–9)	7 (7–8)	0.057
Drinking (lifetime)	514/710 (72%)	185/213 (87%)	34/45 (76%)	0.055	275/419 (66%)	20/33 (61%)	0.56
Drinking habit (the preceding year)	429/710 (60%)	166/213 (78%)	30/45 (67%)	0.11	215/419 (51%)	18/33 (55%)	0.72
Unchanged drinking habit	159/423 (38%)	69/167 (41%)	11/29 (38%)	0.73	77/209 (37%)	2/18 (11%)	0.052
Improved drinking habit	264/423 (62%)	98/167 (59%)	18/29 (62%)	0.73	132/209 (63%)	16/18 (89%)	0.052
AUDIT score	2 (0–5)	4 (1–9)	6 (0–12)	0.72	0 (0–3)	1 (0–2)	0.80
AUDIT score of 8 or more	111/666 (17%)	61/195 (31%)	17/39 (44%)	0.14	32/401 (8.0%)	1/31 (3.2%)	0.54
Smoking (lifetime)	265/693 (38%)	129/209 (62%)	32/44 (73%)	0.17	96/407 (24%)	6/33 (18%)	0.48
Smoking habit (the preceding year)	144/693 (21%)	70/209 (33%)	17/44 (39%)	0.51	54/407 (13%)	3/33 (9.1%)	0.68
Unchanged smoking habit	41/140 (29%)	22/69 (32%)	3/16 (19%)	0.46	16/53 (30%)	0/2	0.89
Improved smoking habit	99/140 (71%)	47/69 (68%)	13/16 (81%)	0.46	37/53 (70%)	2/2	0.89
FTND score	0 (0–0)	0 (0–2)	0 (0–3)	0.57	0 (0–0)	0 (0–0)	0.49
FTND score of 3 or more	92/686 (13%)	48/207 (23%)	11/43 (26%)	0.74	32/404 (7.9%)	1/32 (3.1%)	0.52
K6 score	3 (1–6)	2 (0–5)	3 (1–5)	0.68	3 (1–6)	5 (2–8)	0.12
K6 score of 8 or more	108/694 (16%)	25/210 (12%)	4/45 (8.9%)	0.75	69/405 (17%)	10/34 (29%)	0.071
HADS score	8 (4–13)	8 (4–12)	8 (4–13)	0.49	8 (5–13)	8 (5–15)	0.51
HADS score of 20 or more	40/690 (5.8%)	9/207 (4.3%)	3/45 (6.7%)	0.78	24/404 (5.9%)	4/34 (12%)	0.33

Categorical variables are shown as proportion (%), and continuous variables are shown as median (interquartile range)

*BMI* body mass index; *ASA-PS* the American Society of Anesthesiologists physical status classification; *sAs* Surgical Apgar score; *AUDIT* Alcohol Use Disorders Identification Test; *FTND* Fagerström Test for Nicotine Dependence; *K6* 6-item Kessler Psychological Distress Scale; *HADS* Hospital Anxiety and Depression Scale

*p < 0.05

Table 3 shows the sex- and age-adjusted odds ratio (OR) and 95% confidence interval (CI) of each factor. Anesthesia time (hours) (OR: 1.53 [CI: 1.37–1.73], p < 0.001), sAs (OR: 0.82 [CI: 0.70–0.97], *p* = 0.019), and K6 score (OR: 1.07 [CI: 1.00–1.13], *p* = 0.040) were significant risk factors associated with the development of postoperative complications. Preoperative use of psychotropics were not associated with postoperative complications.

**Table 3:** Sex- and age-adjusted analysis to examine the association of postoperative complications and patient characteristics

	Postoperative complications		M-W U/x^2^ test		
	No (642)	Yes (79)	*p* value	OR (95% CI)	*p* value
BMI (kg/m^2^)	23 (21–25)	23 (21–25)	0.91	0.96 (0.89–1.03)	0.26
ASA-PS I	202	10	0.002*	II/I 1.89 (0.93–4.15)	0.077
ASA-PS II	417	66		III/I 1.37 (0.28–5.18)	0.67
ASA-PS III	23	3		III/II 0.73 (0.17–2.21)	0.61
Preoperative use of psychotropics	74/615 (12%)	11/78 (14%)	0.58	0.95 (0.47–1.93)	0.89
Anesthesia time (minutes. Hours in OR)	169 (127–234)	298 (210–389)	p < 0.001*	1.53 (1.37–1.73)	p < 0.001*
sAs	8 (7–9)	7 (6–8)	0.001*	0.82 (0.70–0.97)	0.019*
Drinking (lifetime)	460/632 (73%)	54/78 (69%)	0.51	0.79 (0.46–1.41)	0.42
No drinking habit (the preceding year)	249/632 (39%)	30/78 (38%)	0.87	0.99 (0.58–1.69)	0.98
Unchanged drinking habit	146/376 (39%)	13/47 (28%)	0.14	0.74 (0.34–1.53)	0.42
Improved drinking habit	230/376 (61%)	34/47 (72%)	0.14	0.74 (0.69–2.20)	0.47
AUDIT score	1 (0–5)	2 (0–8)	0.13	1.23 (0.97–1.07)	0.45
AUDIT score of 8 or more	93/596 (16%)	18/77 (23%)	0.084	1.48 (0.77–2.76)	0.24
Smoking (lifetime)	225/616 (37%)	38/77 (49%)	0.029*	1.39 (0.80–2.39)	0.24
No smoking habit (the preceding year)	492/616 (80%)	57/77 (74%)	0.23	0.68 (0.38–1.27)	0.22
Unchanged smoking habit	38/122 (31%)	3/18 (17%)	0.34	0.79 (0.18–2.46)	0.71
Improved smoking habit	84/122 (69%)	15/18 (83%)	0.34	1.48 (0.74–2.83)	0.26
FTND score	0 (0–0)	0 (0–0)	0.44	1.10 (0.96–1.25)	0.15
FTND score of 3 or more	80/611 (13%)	12/75 (16%)	0.49	0.79 (0.40–1.68)	0.53
K6 score	3 (1–6)	4 (1–6)	0.55	1.07 (1.00–113)	0.040*
K6 score of 8 or more	94/615 (15%)	14/79 (18%)	0.57	1.51 (0.77–2.80)	0.22
HADS score	8 (4–13)	8 (5–14)	0.96	1.02 (0.98–1.06)	0.24
HADS score of 20 or more	33/611 (5.5%)	7/79 (8.9%)	0.22	1.97 (0.76–4.52)	0.15
Length of stay after surgery	8 (5–12)	21 (12–38)	p < 0.001*		

Categorical variables are shown as proportion (%), and continuous variables are shown as median (interquartile range)

*M-W U* Mann–Whitney U test; *x*^*2*^ *test* chi-squared test; *OR* odds ratio; *CI* confidence interval; *BMI* body mass index; *ASA-PS* the American Society of Anesthesiologists physical status classification; *sAs* Surgical Apgar score; *AUDIT* Alcohol Use Disorders Identification Test; *FTND* Fagerström Test for Nicotine Dependence; *K6* 6-item Kessler Psychological Distress Scale; *HADS* Hospital Anxiety and Depression Scale

*p < 0.05

We applied multivariable analysis to examine factors associated with postoperative complications. The correlation between anesthesia time and surgical time, and between the K6 and HADS score, were both 0.6 and higher. As such, only the more highly causal variable from each pair was included in the final model (Model 1–4) (anesthesia time and K6 score, respectively). The significant risk factors for the development of postoperative complications in each model were as follows (Table 4): Model 1: male sex (OR: 1.86 [CI: 1.00–3.49], *p* = 0.049), anesthesia time (hours) (OR: 1.55 [CI: 1.35–1.80], *p* < 0.001), and K6 score (OR: 1.08 [CI: 1.00–1.16], *p* = 0.033); Model 2: old age (for every 10 years) (OR: 1.34 [CI: 1.05–1.74], *p* = 0.020), anesthesia time (hours) (OR: 1.51 [CI: 1.32–1.75], *p* < 0.001), and K6 score (OR: 1.08 [CI: 1.00–1.15], *p* = 0.041); Model 3 (male): anesthesia time (hours) (OR: 1.47 [CI: 1.23–1.77], p < 0.001); Model 4 (female): anesthesia time (hours) (OR: 1.62 [CI: 1.30–2.06], p < 0.001) and K6 score (OR: 1.12 [CI: 1.02–1.23], *p* = 0.017). Improved drinking/smoking habits and cessation of drinking/smoking after surgical decision-making were not associated with postoperative complications.

**Table 4:** Factors associated with postoperative complications (Adjusted multivariable analysis)

	Model 1		Model 2		Model 3 (male)		Model 4 (female)	
	OR (95% CI)	*p* value	OR (95% CI)	*p* value	OR (95% CI)	*p* value	OR (95% CI)	*p* value
Sex (male)	1.86 (1.00–3.49)	0.049*	1.63 (0.85–3.13)	0.14				
Age/10	1.27 (0.99–1.64)	0.054	1.34 (1.05–1.74)	0.020*	1.30 (0.91–1.92)	0.15	1.35 (0.92–2.02)	0.12
BMI (kg/m^2^)	0.96 (0.89–1.04)	0.37	0.99 (0.91–1.07)	0.75	1.03 (0.91–1.16)	0.66	0.93 (0.81–1.04)	0.21
ASA-PS II^a^	1.55 (0.72–3.58)	0.27	1.35 (0.62–3.13)	0.46	1.78 (0.45–11.8)	0.45	1.49 (0.54–4.28)	0.45
ASA-PS III ^a^	1.20 (0.22–5.10)	0.82	0.75 (0.10–3.60)	0.74	0.70 (0.03–9.07)	0.79	1.26 (0.06–10.5)	0.85
sAs	1.10 (0.90–1.38)	0.37	1.09 (0.88–1.37)	0.44	1.08 (0.81–1.47)	0.62	1.10 (0.81–1.57)	0.56
Anesthesia time (hours)	1.55 (1.35–1.80)	p < 0.001*	1.51 (1.32–1.75)	p < 0.001*	1.47 (1.23–1.77)	*p* < 0.001*	1.62 (1.30–2.06)	p < 0.001*
Unchanged drinking habit ^b^	0.93 (0.41–2.07)	0.87						
Improved drinking habit ^b^	0.87 (1.45–1.66)	0.66						
Unchanged smoking habit ^b^	1.51 (0.32–5.18)	0.56						
Improved smoking habit ^b^	0.91 (0.41–1.95)	0.82						
K6 score	1.08 (1.00–1.16)	0.033*	1.08 (1.00–1.15)	0.041*	1.04 (0.93–1.16)	0.52	1.12 (1.02–1.23)	0.017*
AUDIT score			1.00 (0.95–1.06)	0.88	1.00 (0.94–1.06)	0.99	1.02 (0.88–1.13)	0.76
FTND score			1.08 (0.92–1.25)	0.31	1.12 (0.95–1.31)	0.18	0.73 (0.37–1.16)	0.21
R^2^	18%		17%		16%		17%	
AUC	81%		80%		79%		78%	
Accuracy	89%		89%		83%		93%	

*OR* odds ratio; *CI* confidence interval; *BMI* body mass index; *ASA-PS* the American Society of Anesthesiologists physical status classification; *sAs* Surgical Apgar score; *K6* 6-item Kessler Psychological Distress Scale; *AUDIT* Alcohol Use Disorders Identification Test; *FTND* Fagerström Test for Nicotine Dependence; *AUC* area under the curve

^a^Odds ratio of ASA-PS I

^b^Odds ratio of “no drinking/smoking habits for the preceding year leading up to surgical decision-making”

*p < 0.05

## Discussion

This study found that preoperative psychological stress is an independent risk factor for postoperative complications in adult surgical patients. In addition, K6 may be a useful tool for its evaluation.

First, preoperative psychological stress predicted the subsequent occurrence of postoperative complications. This may suggest that being optimistic for the surgery had positive effect on faster recovery, and preoperative intervention against psychological stress may reduce the risk of postoperative complications. In previous studies, preoperative psychological stress or depression has been reported to be associated with postoperative complications and poorer recovery [8, 26]. For example, depression increases postoperative wound infection rates due to decreased immunity [27, 28]. Furthermore, when depression and pain occur simultaneously, depression lowers the pain threshold and worsens both conditions [29, 30]. Pain is an obstacle to early postoperative recovery as it delays patient mobilization, causes an increase in blood pressure, and leads to infections, hemorrhage, and delirium [31]. Since correlations between depression and postoperative complications have been previously reported, psychological stress, which is a precursor to depression, can also cause immunosuppression and aggravation of pain that trigger postoperative complications [32]; thus, perioperative psychological stress may adversely affect postoperative outcomes.

In this study, the K6 was shown to be a convenient and effective tool for evaluating psychological stress. Although no study has yet directly compared the HADS to the K6 or evaluated the association between the K6 score and risks of postoperative complications, the K6 may be useful for simple screening of preoperative psychological stress as elderly patients can also complete in the limited time available on the day before surgery.

Unlike previous studies, our results showed that improved drinking/smoking habits, the AUDIT score (alcohol dependence), and the FTND score (nicotine dependence) were not associated with postoperative complications in multivariable analysis. Assuming that preoperative psychological stress is a risk factor for postoperative complications, this suggests that drinking/smoking cessation can itself be hugely stressful to mental health [33], which may have counteracted any positive results of cessation, such as improving immune function and cardiopulmonary function [34–36]. Additionally, since women made up 64% of all cases in this study, the proportion of patients who smoked within the year leading up to the surgical decision was low (21%), which may explain the discrepancies with previous studies. In fact, mean FTND scores of 3.9 points in Canadian patients [37] and 4.1 points in Chinese patients have been reported in past studies [38]. Regarding AUDIT scores, 6.5 points (median) have been reported in American intensive care unit patients [39], and 7.9 points (mean) were reported in Japanese firefighters [15]. In comparison, the patient group in the present study had lower FTND scores (mean 0.7, median 0) and AUDIT (mean 3.6, median 2). The growing importance of health literacy in surgical populations and smoking/drinking cessation instructions from surgeons also likely contributed to the decline of the influence of smoking and drinking [40]. Moreover, another reason may be that 29% of all surgeries in this study were breast surgery, which has a low rate of postoperative complications, regardless of drinking/smoking habits. However, several studies have reported the long-term benefits of smoking cessation due to surgical decision-making. In this study, the proportion of patients who either reduced or quit their drinking/smoking habits between surgical decision-making and surgery were 62 and 71%, respectively. These results support the belief that surgical decision-making, as a life event, is a highly effective “teachable moment” for lifetime smoking cessation and long-term moderation of drinking habits [41, 42].

Modifiable risk factors were anesthesia (surgical) time and the K6 score (psychological stress) in this study. Therefore, shortening the surgical time is important to reduce postoperative complications, particularly in elderly patients. Psychological support is an emerging area of prehabilitation practice [43]. Although HADS is used, an optimal preoperative screening tool is yet to emerge [4]; K6 may be simpler and more useful for preoperative screening of mental status.

This study had some limitations. First, since this was an observational study, we cannot make any inferences concerning the causal association between psychological stress and postoperative complications. Significant psychological stress observed in preoperative patients may simply be a progression of depressive tendencies that occur as a result of disease progression and announcement of their illness. It is possible that the patient’s condition, which was not assessed by ASA-PS, was already at risk of developing postoperative complications. Second, it is also likely that the number of missing items and recall bias, which are typical issues when using questionnaires, are higher than normal in our hospital given that we treat many elderly patients. Especially in elderly cancer patients, cognitive function is likely to be impaired by chemotherapy, electrolyte and endocrine abnormalities, brain tumors or brain metastases, and malnutrition [44]; therefore, the questions in this survey may have been too many. Hence, at most diagnosis procedure combination hospitals where patients are admitted 1 day before surgery, it may be necessary to shorten the questionnaire by limiting items to those that have the most significant effects on patient outcomes. Third, the effect of recent history of anesthesia and surgery on postoperative outcomes was not included in the analysis. This could affect preoperative mental status; however, we did not ask this question because many patients often misinterpret sedation during neuraxial anesthesia and examinations as general anesthesia with endotracheal intubation. We consider that we were able to assess the impact of surgical experience on preoperative psychological distress to some extent in K6.

In conclusion, our analysis suggests that psychological stress, as evaluated by the K6, may increase the frequency of postoperative complications. Reports on the use of the K6 in the perioperative period have thus far been limited. Future studies should consider K6 while assessing the need for preoperative psychological support or intervention.

## Data Availability

Data from the reported cases will be made available on reasonable request from the corresponding author.

## Abbreviations

AE: Adverse event
ASA-PS: The American Society of Anesthesiologists physical status classification
AUC: Area under the curve
AUDIT: Alcohol Use Disorders Identification Test
BMI: Body mass index
CI: Confidence interval
FTND: Fagerström Test for Nicotine Dependence
HADS: Hospital Anxiety and Depression Scale
JCOG: Japan Clinical Oncology Group
K6: 6-item Kessler Psychological Distress Scale
OR: Odds ratio
sAs: Surgical Apgar score
